# Cognitive Training During Midlife: A Systematic Review and Meta-Analysis

**DOI:** 10.1007/s11065-024-09649-z

**Published:** 2024-09-05

**Authors:** Chen Zhu, Shalini Arunogiri, Qi Li, Elizabeth H. X. Thomas, Caroline Gurvich

**Affiliations:** 1 HER Centre Australia, Department of Psychiatry, School of Translational Medicine, Faculty of Medicine, Nursing and Health Sciences, Monash University, Melbourne, Australia; 2 Eastern Health Clinical School, Faculty of Medicine, Nursing and Health Sciences, Monash University, Turning Point, Melbourne, Australia

**Keywords:** Cognitive training, Cognition, Midlife, Menopause, Far-transfer effect, Near-transfer effect

## Abstract

Midlife has been suggested to be a crucial time to introduce interventions for improving cognitive functions. The effects of cognitive training (CT) in healthy middle-aged populations and more specifically during the menopausal transition have not been systematically investigated. To investigate the effects of CT on cognition in healthy middle-aged adults and specifically in females during the menopause transition, literature was searched inception to July 2023 and studies were included that examined the effects of CT on a defined cognitive outcome. The improvement on cognitive performance following CT was the main outcome measured as mean difference (from baseline to immediate post) estimates with corresponding 95% confidence intervals (CI) in meta-analysis and was discussed with the support of subgroup analysis based on outcome type (i.e., far or near-transfer) and cluster tabulations. Nineteen articles were included in the qualitative synthesis with a total of 7765 individuals, and eight articles were included in the meta-analyses. CT was categorized into six type clusters: Game-based CT, General CT, Speed of Processing Training, Working Memory Training, Strategy-based CT, and Cognitive Remediation. Cognitive outcome was divided into six clusters: working memory, verbal memory, language, executive function, attention/processing speed, and visual memory. Meta-analysis reported significant improvement in the domain of executive function (0.48, 95% CI 0.08–0.87), verbal memory (0.22, 95% CI 0.11–0.33), and working memory (0.16, 95% CI 0.05–0.26). CT confers benefits on various cognitive domains, suggesting a potential role of CT to promote optimal cognitive functioning in the midlife and specifically in women during the menopause transition.

**Supplementary Information:**

The online version contains supplementary material available at 10.1007/s11065-024-09649-z.

## Introduction

Midlife, defined as the age range from 40 to 65 years, emerges as a critical time for introducing interventions aimed at maintaining or improving cognitive functions in individuals susceptible to neurodegenerative diseases (Gates et al., 2019; Ishtiak-Ahmed et al., 2019). For women, midlife coincides with the menopause transition, which is a period of endocrine change associated with varying physiological changes as well as brain changes, often associated with subtle cognitive symptoms (Maki & Weber, 2021). The menopause transition is considered a time of dynamic neurological transition, associated with changes in brain structure, connectivity, and metabolic profiles, and therefore may also be considered a time of vulnerability or potentially opportunity to optimize cognitive health (Mosconi et al., 2021). One potential intervention is cognitive training (CT) and the current study adopted the terminology framework proposed by Mowszowski et al. (2010). Various cognitive-based techniques were categorized into three approaches: CT, cognitive rehabilitation, and cognitive stimulation (Mowszowski et al., 2010). Of these, CT (i.e., involves practice on a range of cognitive-related tasks and skills that were theoretically driven, via either a strategy-based form or a computerized platform) has received increasing attention given its cognitively therapeutic nature and guided delivery method.

CT has been regarded as an easily implemented non-pharmacological preventive intervention to increase or maintain cognitive functioning in individuals with various neurodegenerative diseases, such as mild cognitive impairment and Parkinson’s disease dementia (Orgeta et al., 2020). Its preventive mechanism is believed to influence brain activity and connectivity associated with aging and neurodegenerative diseases (van Balkom et al., 2020). CT has also been well reviewed for promoting cognitive functioning in both older and younger healthy adults (Bonnechère et al., 2020; Nguyen et al., 2021; Scholl et al., 2021; Young et al., 2021). However, limited studies have been conducted to evaluate the benefits of CT to improve cognitive functioning in healthy people in midlife. The potential benefits of CT in midlife, using a broad definition to enable conclusions to be drawn about intervention content, length, and design, has not been previously investigated in a systematic manner.

Therefore, the purpose of this study is to systematically review the studies of CT in healthy people in midlife. In line with the definitions in past research (Gobet & Sala, 2023) and a taxonomy of transfer developed by Barnett and Ceci (2002), this review will further explore the near-transfer (i.e., the training effect of an intervention in one domain on performance in similar domains by utilizing an outcome measurement that shares similar components with the training task) and far-transfer effects (i.e., the training effect of an intervention in one domain on performance in seemingly different domains by utilizing an outcome measurement that does not share similar components with the training task) of CT on individual cognitive domains through meta-analysis. This review will also identify any studies using CT in healthy women in midlife to draw inferences about whether CT may offer some benefits for cognition during menopause transition.

## Methods

### Search Strategy and Eligibility Criteria

**Fig. 1:**
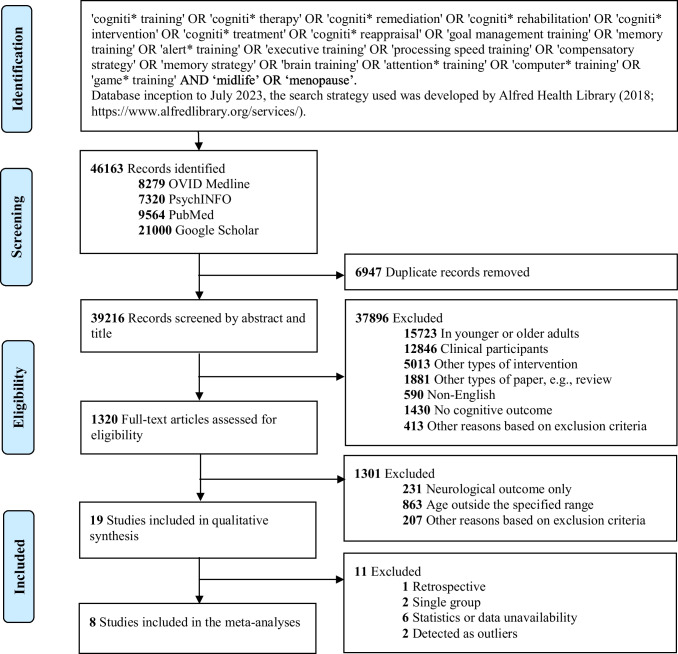
Flow chart of selection process following the PRISMA 2020 guideline

This study was pre-registered on Prospero (CRD42022287617) and followed the guideline of Preferred Reporting Items for Systematic Reviews and Meta-Analyses Statement (PRISMA; Page et al., 2021). The search terms, selection process, and results are shown in Fig. 1. Previous studies were identified by searching the abstracts, keywords, and content, as well as by reviewing the reference lists of identified studies. A full description of inclusion and exclusion criteria is available in eTable 1 in the Supplement. Specifically, the intervention should be any form of CT defined by Mowszowski et al. (2010) that involved repeated practice on standardized exercises or techniques of specified cognitive domain(s) for the purpose of enhancing cognitive function.

### Data Extraction and Quality Assessment

The primary researcher (C.Z.) screened all titles, abstracts, and full-text articles, while a second researcher (C.G.) independently reviewed a random 50% of the screened results to ensure the accuracy and consistency of the screening process. Two researchers (C.Z. and C.G.) independently conducted the data extraction and risk of bias analysis. Two other independent researchers (S.A. and E.T.) reviewed the above stages using a standardized spreadsheet to indicate agreement or disagreement. Discrepancies between researchers were resolved through discussion until consensus was reached. An excellent level of inter-rater agreement, measured by percentage agreement, was achieved, surpassing 85.00% for all stages. The risk of bias (Supplementary eTable 2) of included studies was assessed according to the Revised Cochrane Risk-of-Bias (RoB2; Sterne et al., 2019), Risk of Bias in Non-randomized Studies of Exposure (ROBINS-E; Higgins et al., 2024), and Risk of Bias in Non-randomized Studies of Interventions (ROBINS-I; Sterne et al., 2016). The quality assessment of meta-analyses was evaluated by the GRADEpro Guideline Development Tool (GRADEpro GDT) in accordance with Cochrane and Grading of Recommendations, Assessment, Development, and Evaluations (GRADE) guideline (Higgins et al., 2019; Schünemann et al., 2013).

### *Syntheses and Statistical Analysis*

All the included studies were qualitatively synthesized by cluster identification based on training types and cognitive outcomes through tabulations. Eligible studies were included in the meta-analysis and meta-regression.

The meta-analysis and meta-regression were performed through *meta* (Balduzzi et al., 2019) and *metafor* (Viechtbauer, 2010) packages in R (Version 4.2.3; R Core Team, 2023). Randomized clinical trials (RCTs) and cross-over studies were included in the analysis to explore the efficacy of CT on each cognitive domain identified in the qualitative synthesis. In the cross-over studies, only the data from the first-time interval (i.e., from baseline to the cross-over point) was included in the meta-analysis to ensure that the targeted training was only delivered in the experimental group. Sub-group analysis based on the type of cognitive outcome (i.e., far-transfer and near-transfer) was conducted if the study number in each cognitive domain allowed. The relationship of training outcome with training length and number of sessions was further explored through meta-regression.

The standardized mean difference (SMD) was calculated as the estimated mean difference between baseline (pre-training assessment) and immediate post (immediate assessment after training with no significant time interval as defined by authors in the included studies) and used as the effect measure in the meta-analyses. Correlation coefficient was set at 0.7 for standard deviation (SD) of SMD calculation if the value was not able to be extracted from the primary papers or accessed from the research group (Higgins et al., 2019). Both the common and random effect models were conducted to estimate the pooled SMD. Cochran’s *Q* tests and the *I*^*2*^ statistic were used to evaluate the statistical and proportional significance of heterogeneity. The significance level was set at < 0.05. *Dmetar* package was used to identify the outliers. Funnel plots were used to assess the publication bias of meta-analyses.

## Results

### Study and Training Characteristics

Nineteen articles were included in the qualitative synthesis: 15 of these were primary studies (Ackerman et al., 2010; Anderson et al., 2013; Ballantyne et al., 2021; Bonnechère et al., 2021; Chapman et al., 2015; Corbett et al., 2015; Emch et al., 2019; Felton et al., 2019; George et al., 2020; McLaughlin et al., 2018; Mridula et al., 2017; Namratha et al., 2017; Pang & Kim, 2021; Unkenstein et al., 2017; Wolinsky et al., 2013) and four (Anderson et al., 2014; Roheger et al., 2020a, b; Wolinsky et al., 2016) were either a follow-up study or further analysis of a previous study or data. Three of the primary studies focused specifically on a female menopausal population (Ballantyne et al., 2021; Pang & Kim, 2021; Unkenstein et al., 2017). Table 1 provides the basic characteristics of study design and population included in the qualitative synthesis. Detailed information of cognitive training type, definition, duration, assessment time points, and content are summarized in Table 2. Five of the primary studies included a follow-up assessment to evaluate the maintenance effect (Anderson et al., 2013; Ballantyne et al., 2021; George et al., 2020; Unkenstein et al., 2017; Wolinsky et al., 2013), with only one study incorporating a single booster session (Wolinsky et al., 2013). The CT included in this systematic review was categorized into six clusters based on the delivery platform and techniques involved: Game-based CT (Ackerman et al., 2010; Bonnechère et al., 2021; McLaughlin et al., 2018), General CT (Anderson et al., 2013, 2014; Corbett et al., 2015; Roheger et al., 2020a, b), Speed of Processing Training (Wolinsky et al., 2013, 2016), Working Memory Training (Emch et al., 2019; Felton et al., 2019; George et al., 2020; Mridula et al., 2017; Namratha et al., 2017), Strategy-based CT (Chapman et al., 2015; Pang & Kim, 2021; Unkenstein et al., 2017), and Cognitive Remediation (Ballantyne et al., 2021).

**Table 1:** Characteristics of included studies in the preliminary synthesis

Author, year	Trial type	Study group	Group details		Participants number	Gender (%)		Age		Population health-related characteristics
						Male	Female	Mean^a^	SD/range^b^	
Ackerman et al., 2010	Cross-over	Group1	Training → Active Control		39	53.85%	46.15%	60.7	50–71	Community middle-aged adults
		Group2	Active Control → Training		39					
Anderson et al., 2013 Anderson et al., 2014	RCTs	Group1	Training		30	46.67%	53.33%	62.3	3.4	Community older adults screened for dementia and psychiatric history
		Group2	Active Control		32	43.75%	56.25%	63.6	4.1	
Bonnechère et al., 2021	Retrospective		Training		12,000	NA	NA	NA	60–64	Community middle to older aged adults
Chapman et al., 2015	RCTs	Group1	Training		18	44.44%	55.56%	61.8	3.3	Cognitively normal adults screened for dementia, psychiatric history, depression with good general health assessed by physician
		Group2	Wait-list Control		19	26.32%	73.68%	64.0	3.6	
Corbett et al., 2015 Roheger et al., 2020a; b	RCTs, double-blind	Group1	Training Condition1	6 weeks training	2431	31.10%	68.90%	59.1	6.4	Adults older than 50
				3 months training	2361	38.00%	62.00%	59.1	6.4	
				6 months training	428	25.00%	75.00%	60.19	6.6	
		Group2	Training Condition2	6 weeks training	2556	31.50%	68.50%	58.5	6.5	
				3 months training	2513	31.30%	68.70%	58.2	6.5	
				6 months training	595	28.60%	71.40%	59.03	6.44	
		Group3	Active Control	6 weeks training	1753	37.60%	62.40%	59.1	6.6	
				3 months training	1682	31.20%	68.80%	59.1	6.6	
				6 months training	176	44.30%	55.70%	60.81	7.24	
Felton et al., 2019	RCTs	Group1	Training		74	40.50%	59.50%	50.0	5.8	Recruited from medically underserved area; 60% are homeless; no substance use disorder or psychotic symptoms
		Group2	Active Control		52	48.70%	51.30%	51.2	5.7	
McLaughlin et al., 2018	Cross-over	Group1	Training → Active Control		7	28.57%	71.43%	52.7	2.4	Community middle-aged adults with no history of neurological or psychiatric illness, head injury, or substance abuse; no presence of an untreated sleep disorder; not taking medication(s) known to impact cognition
		Group2	Active Control → Training		7	42.86%	57.14%	52.1	3.4	
Namratha et al., 2017	RCTs	Group1	Training Condition1		15	20.00%	80.00%	51.0	7.43	Middle-aged adults screened for psychological problems and severe sensory deficits
		Group2	Training Condition2		15	33.33%	66.67%	52.6	7.65	
		Group3	Passive Control		15	26.67%	73.33%	52.5	6.05	
Mridula, et al., 2017	RCTs	Group1	Training		15	33.33%	66.67%	52	7.47	
		Group2	Passive Control		15	26.67%	73.33%	52.47	6.0	
George et al., 2020	RCTs	Group1	Training		31	29.03%	70.97%	51.74	7.28	
		Group2	Passive Control		31	32.26%	67.74%	51.61	6.57	
Emch, et al., 2019	RCTs	Group1	Training		30	50.00%	50.00%	55.8	4.3	Community middle-aged adults with no psychiatric disorder, presence of metal in the body, depression, and clinically relevant alterations in brain structure
		Group2	Active Control		27	48.15%	51.85%	55.92	4.25	
Wolinsky et al., 2013 Wolinsky et al., 2016	RCTs	Group1	Training Condition1		98	33.70%	66.30%	57.9	NA	Middle-aged and older adults
		Group2	Training Condition2		83	30.10%	69.90%	56.8	NA	
		Group3	Training Condition3		111	31.50%	68.50%	57.2	NA	
		Group4	Active Control		121	37.20%	62.80%	57.0	NA	
Unkenstein et al., 2017	Single case, longitudinal		Training		32	0	100%	55.0	47–60	Menopausal women recruited from outpatient at hospital with no surgical menopause, chemotherapy-induced menopause, or a history of drug or alcohol addiction, head trauma, or brain disorder
Pang & Kim, 2021	Quasi-experimental	Group1	Training Condition1		18	0	100%	60.9	6.62	Menopausal women over 50 years old with normal cognitive functioning and no presence of depression
		Group2	Training Condition2		12	0		59.42	5.16	
		Group3	Passive Control		12	0		59.33	6.54	
Ballantyne et al., 2021	Single case, repeated measures		Training		27	0	100%	53.7	4.14	Peri/postmenopausal women referred by GP or recruited from community with cognitive complaints and no psychiatric/substance use disorder and suicidal ideation

*RCTs*, randomized control trials; *SD*, standard deviation; *NA*, not available

^a^The values extracted from original studies

^b^Depends on which index is available in the original study

**Table 2:** Descriptions of cognitive training used in the included studies in preliminary synthesis

Training type clusters	Author, year			Cognitive training			
		Intervention	Platform	Site	Training duration	Assessment time point	Content and paradigm
Game-based Cognitive Training (Game-based CT):* Training task delivered on a gameplay basis*	Ackerman et al., 2010	Wii Practice Assignment	Handheld game console, *Nintendo DS* gaming system and *Big Brain Academy* software	Remote and at home	1 h per session, 5 sessions/week, for 4 weeks	T1 = Baseline T2 = Interim T3 = Immediate post	The *Big Brain Academy* game consists of a set of 15 mini-games designed to provide training and practice on a variety of mental tasks
		Reading Assignment	Paper-based				Four packets of newspaper and magazine articles on the topics of medical drugs, food, going green, and technology
	McLaughlin et al., 2018	Brain Training Game Playing	Handheld game console, *Nintendo DS* gaming system and *Big Brain Academy* software	Remote and at home	1 h per session, 3 h/week, 6 weeks, 18 sessions	T1 = Baseline T2 = Interim T3 = Immediate post	*Big Brain Academy consists of 15 activities grouped into five categories (think, memorize, analyze, compute, and identify) designed to stimulate/train and practice mental abilities. Activities required various cognitive skills, including simple attention and working memory, perceptual reasoning, and visuospatial skills*
		Answering Trivia Questions	Computerized, online				Participants answered a set of randomly generated trivia questions on a variety of topics, including music, history, science, art, and literature
	Bonnechère et al., 2021	Cognitive Mobile Games	Mobile-based, *Peak brain training*	Remote/home	100 sessions	Over 100 sessions	A set of seven individual short CMG:* Square Numbers, Memory Sweep, Word Pair, Babble Bots, Must Sort, Unique, Rush Back*
General Cognitive Training (General CT): *Task involving multiple cognitive domains*	Anderson et al., 2013 Anderson et al., 2014	Auditory-based Cognitive Training	Computerized, Brain Fitness program (Posit Science, San Francisco, CA)	Remote/home	1 h per day, 5 days per week, for 8 weeks	T1 = Baseline T2 = Immediate post T3 = 6-month follow-up	Consisting of six modules designed to increase the speed and accuracy of auditory processing: (1) time-order judgment of frequency-modulated sweeps, (2) discrimination between pairs of confusable syllables, (3) recognizing sequences of confusable syllables and words, (4) matching pairs of confusable syllables and words, (5) implementing sequences of commands, and (6) answering questions from stories
		Auditory Activities	Computerized				Participants watched educational DVDs on topics including art, history, and literature and answered questions about the content, and the training was non-adaptive
	Corbett et al., 2015 Roheger et al., 2020a; b	General Cognitive Training	Computerized, online	Remote/home	10 min per day, 6 weeks, 3 months, or 6 months, 6 sessions in total	T1 = Baseline T2 = 6-week training T3 = 3-month training T4 = 6-month training	The GCT consisted of six cognitive tasks that trained attention, memory, mathematics, and visuospatial abilities
		Reasoning Cognitive Training					ReaCT focused on 3 reasoning tasks and 3 problem-solving tasks
		Equivalent Internet-based Tasks					The control group performed a game in which people were asked to put a series of statements in correct numerical order
Working Memory Training (WMT): *Task involving single domain of working memory*	Felton et al., 2019	Working Memory Training	Computerized, *PSSCogReHab, Psychological Software Services*	Remote/home	30 min per session, 3 days per week for 5 to 7 weeks, 15 sessions	T1 = Baseline T2 = Immediate post	The active training program in which participants completed four modules during each session: Sequence Recall of Digits—Auditory, Sequence Recall of Reversed Digits—Auditory, Sequenced Recall of Words—Visual, Verbal Memory—Categorizing
		Active Control Training	Computerized, online				The control training condition was designed to utilize the same essential features of the active training including the stimulus, response, and feedback, without engaging working memory
	Namratha et al., 2017 Mridula et al., 2017 George et al., 2020	Working Memory Training	Computerized, *Paradigm software version 2.5.0.68*	On-site	45–60 min per session, 2 sessions per week, 10 sessions over 5 weeks	T1 = Baseline T2 = Immediate post T3 = 3-month follow-up	The working memory training comprised of six working memory tasks: semantic sorting, double digit ordering, multistage problem solving, sentence ordering, N-back tasks, and verbal fluency tasks
	Emch et al., 2019	Adaptive Working Memory Training	Computerized, *Inquisit 5*	At-home	4 sessions per week, 32 sessions over 8 weeks	T1 = Baseline T2 = Immediate post	The training of the experimental group was based on an adaptive online n-back (both verbal and visual) paradigm comprising 9 blocks per session
		Low-level Working Memory Training					A low-level working memory training (i.e., stable level of verbal 1-back task)
Speed of Processing Training (SPT): *Task involving single domain of processing speed*	Wolinsky et al., 2013 Wolinsky et al., 2016	Visual Speed of Processing Program	Computerized, *Double Decision*	On-site	10 h in 5–6 weeks	T1 = Baseline T2 = 1-year follow-up	SOPT program was originally named *Road Tour* and delivered from a CD platform when used in this study, but subsequently named *Double Decision* and moved to a web-based platform
				On-site	10 h in 5–6 weeks + 4 h booster at 11 months post-baseline		
				At-home	10 h in 5–6 weeks		
		Attention Control Program	Computerized, *Boatload of Puzzles*	On-site	10 h in 5–6 weeks		Crossword puzzles
Strategy-based Cognitive Training (Strategy-based CT): *Guided practice in compensatory or adaptive strategies*	Pang & Kim, 2021	Calendar Training	Smartphone-based, *One Day Calendar (Korea)*	Remote/home	12 weeks	T1 = Baseline T2 = Immediate post	Three sections were involved (1) appointments, (2) items to be completed, and (3) journaling. In the appointment section, participants recorded tasks that needed to be performed at specific times. In the items to be completed section, participants created a to-do list without indicating when the items were to be completed. In the journaling section, participants wrote about important events that happened to them that day
	Unkenstein et al., 2017	The LaTCH Memory Strategies Program	Manualized and interactive courses	On-site, in group	2-h session per week, 4 weeks	T1 = 1-month before baseline T2 = Baseline T3 = Immediate post T4 = 3-month follow-up	Provided information about memory stores and processes and changes in memory related to health and lifestyle issues. Common everyday memory problems were discussed and memory strategies for a variety of tasks and situations were introduced and practiced. The group addressed emotional reactions to memory challenges and strategies to develop self-confidence and to promote healthy brain ageing. Handouts covering session material were provided at the end
	Chapman et al., 2015	Gist Reasoning Training	Didactics and practices	On-site, in group and individually at home without supervision	1-h session per week, for 12 weeks; 2 additional 1 h sessions per week, for 12 weeks	T1 = Baseline T2 = Interim T3 = Immediate post	Gist reasoning training is strategy-based rather than content specific and entails a systematic use of 3 cognitive processes including strategic attention, integrated reasoning, and innovation to process all types of data. The gist reasoning training involved top-down cognitive control of complex information that is maintained, manipulated and synthesized into abstracted meanings. Training also involved practice of innovative thinking by generating diverse interpretations as well as a wide variety of ways to approach or solve a task at hand, whether work or leisure related
Cognitive Remediation (CR): *Targeting at individual areas of weakness in daily functioning and implementing strategies to improve or compensate for these difficulties*	Ballantyne et al., 2021	Cognitive Remediation Intervention	Didactics and group discussion	On-site, in group	2-h session per week, 5 weeks plus booster session 1 month after	T1 = Baseline T2 = Immediate post T3 = 1-month follow-up	Each session consisted of didactics, group discussion about the material, and goal setting. Cognitive compensatory strategies were incorporated into each session. Given the potential role of medical factors in cognitive decline, relevant lifestyle strategies were also discussed in relation to the targeted area of cognition. Each session was accompanied by a handout which summarized the psychoeducational information and relevant worksheets

Of the 19 articles, 11 were excluded from the meta-analyses according to the eligibility criteria defined in eTable 1 relating to single-case design, data unavailability at immediate post-training time point, and outliers. Eight articles covering Game-based CT, General CT, Strategy-based CT, and Working Memory Training were included in the final meta-analyses.

### Cognitive Outcomes

Cognitive outcomes were categorized into six domain clusters in this systematic review, which were working memory, verbal memory, executive function, language, attention/processing speed, and visual memory. While working memory is often conceptualized as an executive function, it was separated into its own domain in the current review as several studies included specific training on working memory and its benefits. Therefore, keeping working memory as its own domain provides an avenue to separately examine the working memory domain, distinct from the broader umbrella notion of executive functions. The definitions of each cognitive outcome and results summary from qualitative synthesis are displayed in Table 3. The categorization of near- and far-transfer of cognitive outcomes in this review adhered to the classifications determined by the authors in the included studies. In cases where the transfer effects were not explicitly specified by the included studies, categorization was based on an assessment of the difference between training content and outcome measures, following the taxonomy of transfer effect defined by Barnett and Ceci (2002). The generalizability of the effect of CT on broader health-related outcomes (e.g., mood) was also included in Table 3 as far-transfer outcomes.

**Table 3:** Results summary from qualitative synthesis based on training type x cognitive domain x outcome type and far transfers on general outcomes

Training outcomes clusters		Training type clusters					
	Outcome type	General CT	Strategy-based CT	Game-based CT	Working Memory Training	Speed of Processing Training^a^	Cognitive Remediation^b^
Executive Function:* Ability to select and monitor behaviors that facilitate the attainment of chosen goals*	Far transfer	↑	↑	NA	↑	↑	-, ⇑^a^
	Near transfer	↑	↑	↑	NA	NA	NA
Attention/Processing Speed: *Ability to identify, discriminate, integrate, make a decision about information, and to respond to information that normally involves simple attention*	Far transfer	NA	-^b^	NA	NA	↑	-, ⇑^a^
	Near transfer	↑^a^	NA	-	↑	NA	NA
Language: *Language ability including lexical knowledge and lexical retrieval ability*	Far transfer	NA	↑^b^	NA	NA	-	-
	Near transfer	NA	NA	-	NA	NA	NA
Verbal Memory: *Ability to encode, freely recall or recognize verbal information*	Far transfer	↑	-, ⇑^ab^	NA	NA	NA	-, ⇑^a^
	Near transfer	NA	NA	-	↑^a^	NA	NA
Visual Memory: *Ability to process and retain information in a visual format*	Far transfer	NA	NA	NA	NA	NA	-
	Near transfer	NA	NA	-	NA	NA	NA
Working Memory:* Capacity to temporarily hold and mentally manipulate information*	Far transfer	↑	-	NA	NA	NA	-
	Near transfer	↑	NA	-	↑^a^	NA	NA
General Outcomes: *Mood, self-esteem, and menopausal symptoms*	Far transfer	NA	-	⇑	NA	NA	-

↑: Improvement on objective cognitive outcome immediately following training

⇑: Improvement on subjective (i.e., self-reported) cognitive outcome or general outcome (e.g., mood) immediately following training

-: No improvement on tested outcome immediately following training

*NA*, not applicable; *CT*, cognitive training

^a^Maintenance effect (i.e., when the improvement was maintained at a follow-up assessment following the end of training) identified

^b^Results identified in menopausal population

**Fig. 2:**
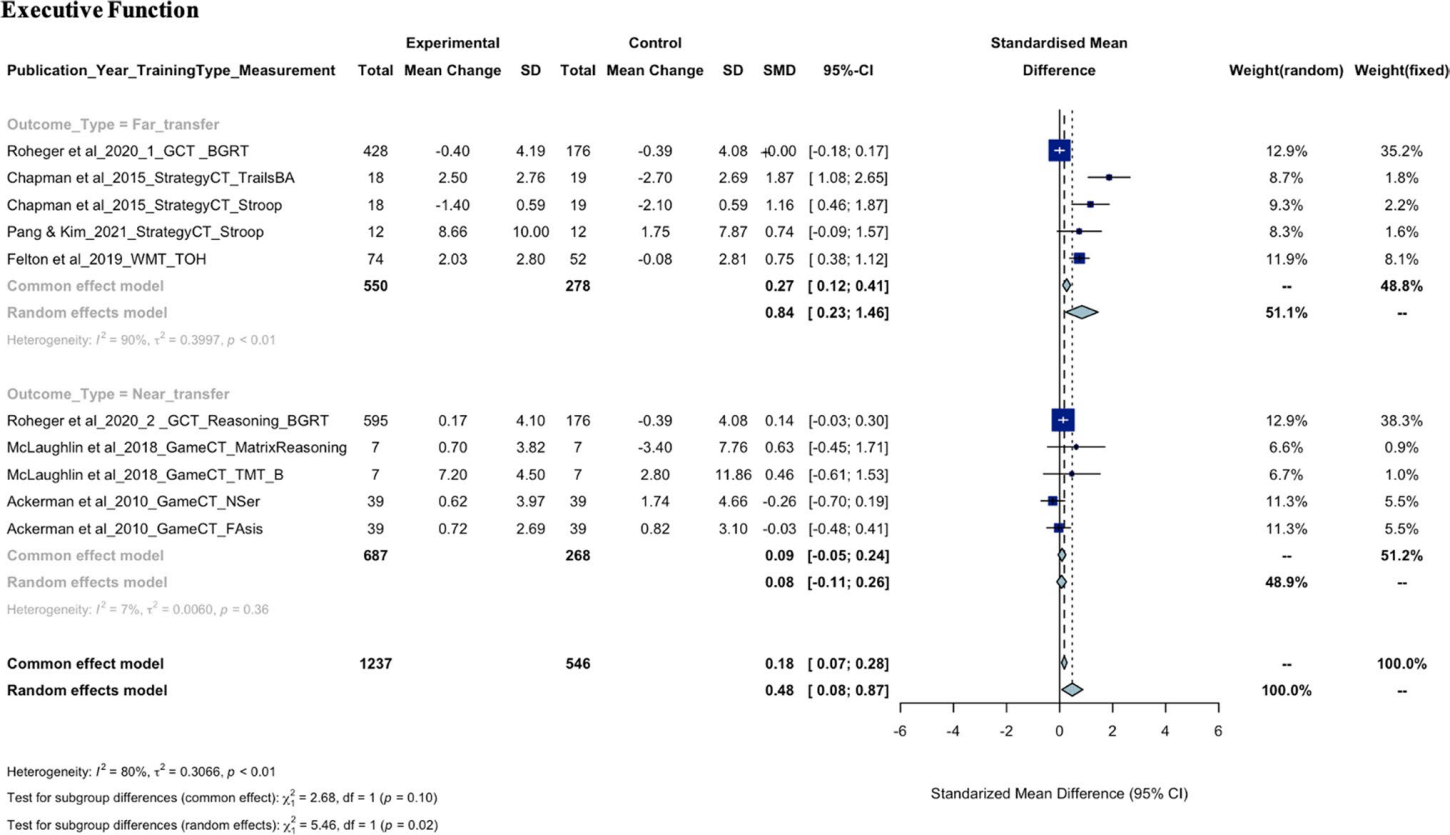

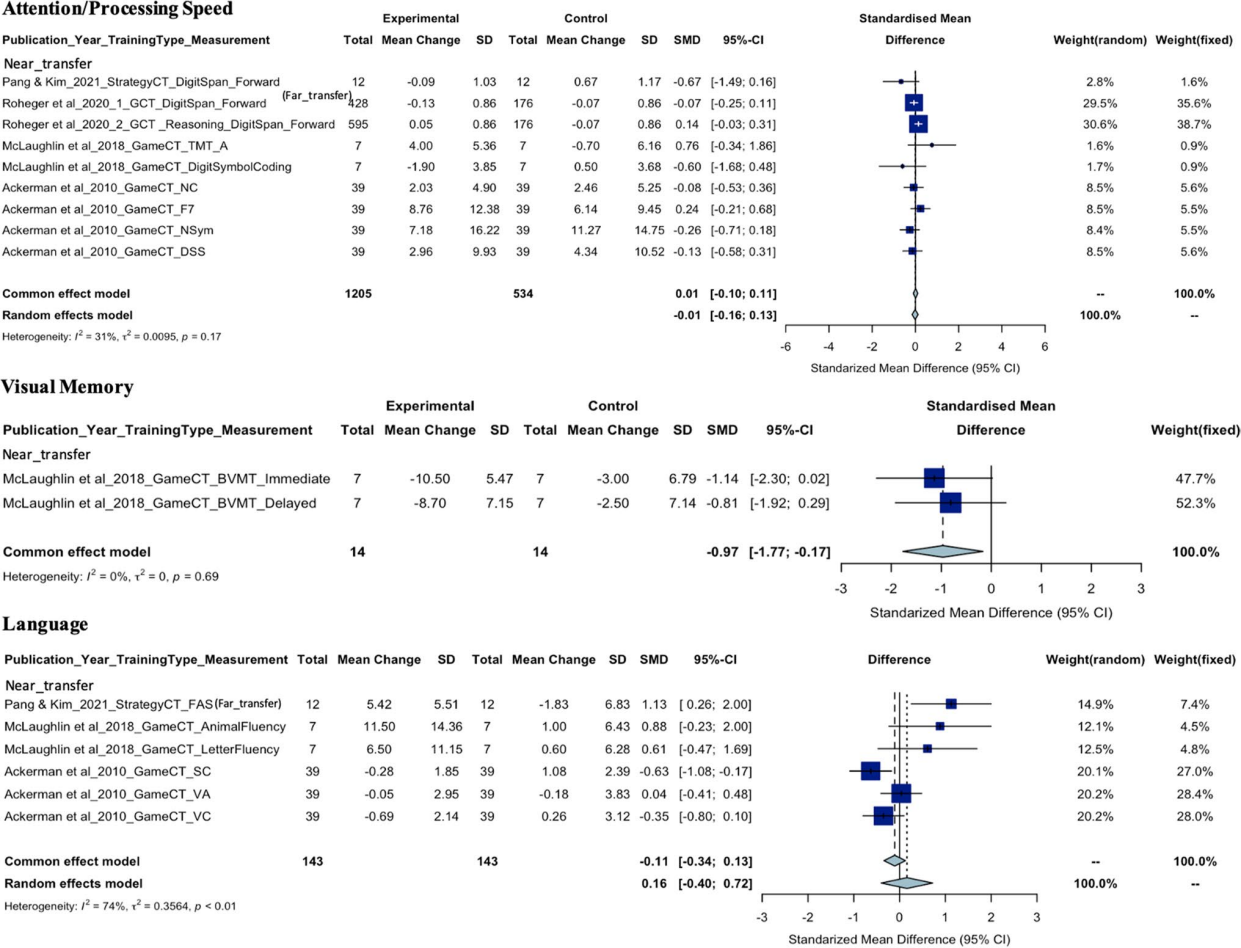

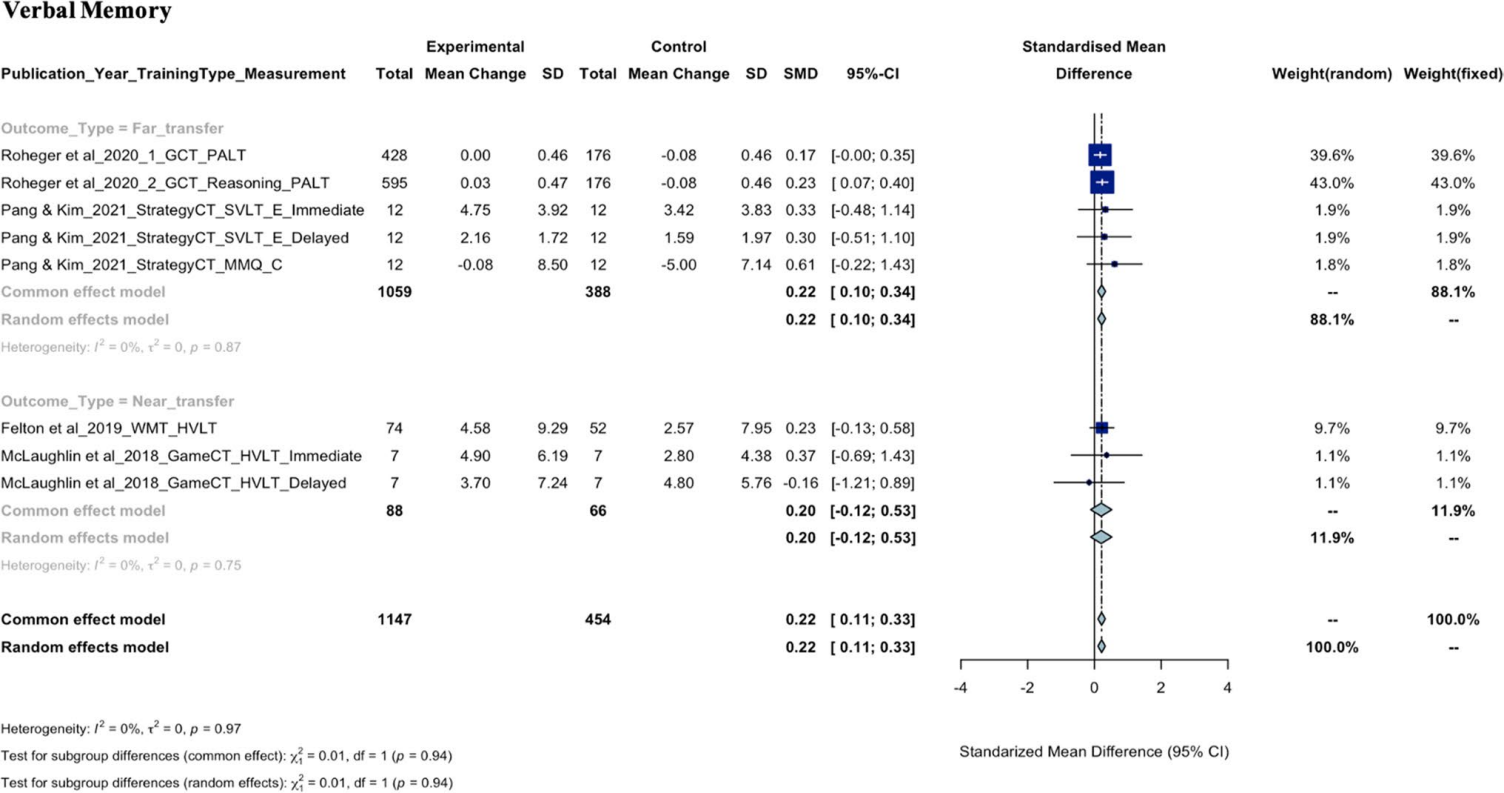

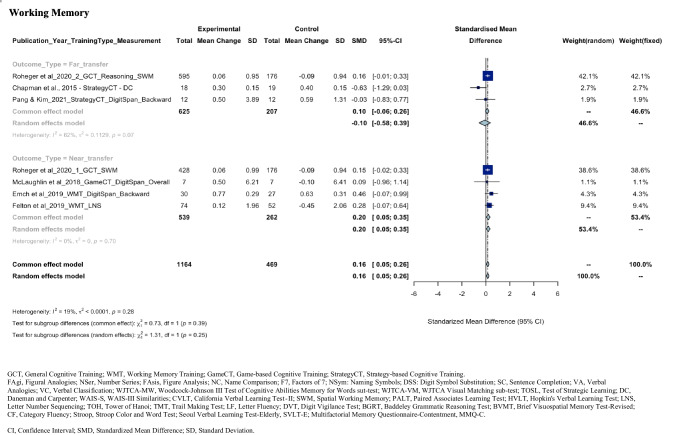
Forest plots of SMD with 95% CI of the meta-analysis of the training effect on each cognitive domain. Subgroup analysis was based on the outcome type

As seen in the meta-analysis findings for each cognitive domain shown in Fig. 2, there is a wide range of neuropsychological tasks used to measure each cognitive domain, requiring careful consideration of heterogeneity statistics. For example, executive function was measured using nine different neuropsychological measures, each assessing different aspects of executive function, such as the Trails B task (divided attention), the Tower of Hanoi (planning), the Stroop task (inhibitory control), and the Baddeley Grammatical Reasoning Test (reasoning). The meta-analysis revealed a high level of heterogeneity (*I*^2^ = 80.5%) even after removing outliers. In contrast, verbal memory, which was consistently measured by word paired learning tasks, showed a very low level of heterogeneity (*I*^2^ = 0.00%). This suggests that a considerable proportion of the variability in effect estimates may be due to differences between outcome measures. The funnel plots for each cognitive domain used to assess the publication bias were shown in eFigure 1. The certainty of evidence according to the GRADE certainty tool suggested low to moderate certainty of evidence for the executive function, language, verbal memory, visual memory, and attention/processing speed as shown in eTable 3.

#### Executive Function

From the meta-analysis, the SMD of 10 comparisons from seven articles was 0.48 (95% CI, 0.08 to 0.87; *P* = 0.02) showing a statistically significant improvement in executive function in experimental groups following CT as compared to control groups who did not receive any training or received non-cognitive intervention. However, there was a significant heterogeneity identified (*Q* = 46.12, *df* = 9, *P* < 0.001;* τ*^2^ = 0.31, *I*^*2*^ = 80.5%) after the removal of outliers (Ackerman et al., 2010; Chapman et al., 2015). The subgroup analysis stratified by outcome type (i.e., far- or near-transfer) revealed a statistically significant difference (χ^2^ = 5.46; *P* = 0.02) in executive function improvement, where far-transfer improvement showed a greater effect 0.84 (95% CI, 0.23 to 1.46; *P* < 0.01). From qualitative synthesis, 11 articles (Ackerman et al., 2010; Ballantyne et al., 2021; Chapman et al., 2015; Corbett et al., 2015; Felton et al., 2019; McLaughlin et al., 2018; Pang & Kim, 2021; Roheger et al., 2020a, b; Wolinsky et al., 2013, 2016) looked at the effect of CT on executive function and revealed improvements on executive function measures following Strategy-based CT and General CT as both far- and near-transfer outcomes, following Working Memory Training and Speed of Processing Training as far-transfer outcomes, and following Game-based CT as near-transfer outcomes in healthy midlife. There was no improvement on neuropsychological measures of executive function following Cognitive Remediation, although there was an improvement on a subjective self-report measure of executive function and this study was specifically conducted in a female, menopause group.

#### Attention/Processing Speed

The meta-analysis included five articles with a total of 1205 individuals in the experimental group and 534 in the control group and the overall pooled estimate was 0.01 (95% CI, − 0.10 to 0.11;* P* = 0.90) showing no significant difference in the attention/processing speed performance domain between experimental groups following CT and controls. Heterogeneity (*Q* = 11.64, *df* = 8, *P* = 0.17;* τ*^2^ = 0.01, *I*^*2*^ = 31.3%) was found not significant after the removal of three outliers (Anderson et al., 2013, 2014; Emch et al., 2019). Twelve articles (Ackerman et al., 2010; Anderson et al., 2013, 2014; Ballantyne et al., 2021; Corbett et al., 2015; Emch et al., 2019; McLaughlin et al., 2018; Pang & Kim, 2021; Roheger et al., 2020a, b; Wolinsky et al., 2013, 2016) from qualitative synthesis investigated the effect of CT on attention/processing speed and revealed near-transfer improvements on attention/processing speed measures following General CT and Working Memory Training. Far-transfer improvements were only found when following Speed of Processing Training as indicated in two articles (that were both related to a same primary study). There was no improvement in attention/processing speed measures following Strategy-based CT or Game-based CT in healthy middle-aged adults. Improvement was only found when attention/processing speed was measured subjectively but not objectively following Cognitive Remediation in the menopausal population.

#### Language

The meta-analysis included three articles involving six comparisons, with a total of 143 individuals in each group. The overall pooled estimate was 0.16 (95% CI, − 0.40 to 0.72; *P* = 0.57) indicating no significant difference in language performance between experimental groups following CT and controls. Significant heterogeneity (*Q* = 19.04, *df* = 5, *P* = 0.002;* τ*^2^ = 0.36, *I*^*2*^ = 73.7%) was identified but no outliers were detected. From qualitative synthesis, six articles (Ackerman et al., 2010; Ballantyne et al., 2021; McLaughlin et al., 2018; Pang & Kim, 2021; Wolinsky et al., 2013, 2016) looked at the effect of CT on language. One article reported far-transfer improvements on language following Strategy-based CT. Five articles reported no improvement on language following Game-based CT, Cognitive Remediation, and Speed of Processing Training.

#### Verbal Memory

As indicated by the meta-analysis, the pooled SMD of eight comparisons from five articles, with a total of 1147 individuals in the experimental group and 454 in the control group, was 0.22 (95% CI, 0.11 to 0.33; *P* < 0.001). This demonstrates a statistically significant improvement in verbal memory in experimental groups following CT than controls. After the removal of outlier (Chapman et al., 2015), heterogeneity was not significant (*Q* = 1.83, *df* = 7, *P* = 0.97;* τ*^2^ = 0.00, *I*^*2*^ = 0.00%). The subgroup difference stratified by outcome type (i.e., near- or far-transfer) was not statistically significant (χ^2^ = 0.01, *P* = 0.94). Qualitative synthesis from twelve articles (Ballantyne et al., 2021; Chapman et al., 2015; Corbett et al., 2015; Felton et al., 2019; George et al., 2020; McLaughlin et al., 2018; Mridula et al., 2017; Namratha et al., 2017; Pang & Kim, 2021; Roheger et al., 2020a, b; Unkenstein et al., 2017) revealed near-transfer improvement on verbal memory following Working Memory Training but not Game-based CT. In terms of far-transfer outcomes, significant improvements on verbal memory tasks were only reported following General CT but not Strategy-based CT. In studies specifically looking at a menopause sample, Strategy-based CT and Cognitive Remediation were associated with improvements in subjective, self-reported verbal memory but not objective tasks.

#### Visual Memory

The meta-analysis of two comparisons within one article, with 14 individuals in each group, revealed an overall pooled estimate of − 0.97 (95% CI, − 1.77 to − 0.17; *P* = 0.02), indicating a significant decrease in visual memory performance following CT. No significant heterogeneity (*Q* = 0.16, *df* = 1, *P* = 0.69;* τ*^2^ = 0.00, *I*^*2*^ = 0.00%) and outliers were found. Qualitative synthesis of two articles (Ballantyne et al., 2021; McLaughlin et al., 2018) that examined the effect of CT on visual memory found no improvement on visual memory following Game-based CT. One article that specifically looked at menopausal women did not find any significant advantage or improvement following Cognitive Remediation on visual memory.

#### Working Memory

The pooled SMD from the meta-analysis of seven comparisons from seven articles, with 1164 participants in experimental and 469 in control, was 0.16 (95% CI, 0.05 to 0.26; *P* = 0.005), revealing a statistically significant improvement in working memory in the experimental groups following CT, as compared to control groups. After the removal of three outliers (Anderson et al., 2013, 2014; Chapman et al., 2015), the level of heterogeneity was not significant (*Q* = 7.41, *df* = 6, *P* = 0.28;* τ*^2^ = 0.00, *I*^*2*^ = 19.1%). The overall test for subgroup difference was not statistically significant (χ^2^ = 0.73, *P* = 0.39). The qualitative synthesis included 14 articles (Anderson et al., 2013, 2014; Ballantyne et al., 2021; Chapman et al., 2015; Corbett et al., 2015; Emch et al., 2019; Felton et al., 2019; George et al., 2020; McLaughlin et al., 2018; Mridula et al., 2017; Namratha et al., 2017; Pang & Kim, 2021; Roheger et al., 2020a, b) that looked at the effect of CT on working memory. Significant improvements on measures of working memory following General CT were reported as either far- or near-transfer. A significant improvement in working memory was also reported following Working Memory Training, described as near-transfer outcome. Strategy-based CT (one in a menopausal population and one in middle-aged adults), Cognitive Remediation (one in a menopausal population), and Game-based CT (one in middle-aged adults) were not associated with significant improvements on working memory.

### Sensitivity Analysis

A meta-regression examining the number of training sessions and training length (in weeks) indicated that a greater improvement in language was significantly associated with longer training length (ranged from 4 to 12 weeks; coefficient = 0.19; 95% CI, 0.06 to 0.33; SE = 0.07; *P* = 0.005), but less training sessions (ranged from 18 to 20 sessions; coefficient =  − 0.53; 95% CI, − 0.98 to − 0.07; SE = 0.23; *P* = 0.02). No significant result was identified from meta-regression for other cognitive domains.

## Discussion

This study investigated the effects of various types of CT on multiple cognitive domains in a healthy middle-aged population through meta-analysis and qualitative synthesis, as well as specific synthesis of the evidence available for the effects of CT for women during the menopause transition.

### Overall Effect of CT on Cognition

Meta-analysis and qualitative synthesis indicated significant benefits on executive function as far-outcomes, that is, when the CT did not specifically involve an executive function task (Felton et al., 2019; Wolinsky et al., 2013, 2016). The results are consistent with previous studies in healthy older adults (Brehmer et al., 2012; Nouchi et al., 2012). Executive function has been widely defined as a domain that encompasses a top-down process that incorporates various cognitive abilities, including inhibitory control, complex attention, and information manipulation. The improvements on lower order cognitive skills, such as processing speed and working memory, may enable adults to make better use of their executive function skills, and thus make executive function more responsive to far-transfer gains through CT. Strategy-based CT, involving the practices of non-specific strategies and techniques (e.g., innovative thinking and strategic attention), showed greater benefits on executive function tasks than General CT and Game-based CT that encompasses various direct training tasks/games on executive function which support the idea that executive function skills can improve with training of broader strategies.

Meta-analyses indicated significant benefits of CT on the domains of verbal memory and working memory in a healthy middle-aged population. Results are consistent with previous studies in healthy younger and older adults suggesting that CT may be particularly effective for healthy adults to improve cognitive functions that are associated with information holding, manipulation, and encoding (Bailey et al., 2014; Ballesteros et al., 2014; Brehmer et al., 2012; Kim, Lee et al., 2022; H. K. Lee et al., 2020; Rosi et al., 2018). Seventy percent of the studies included in the meta-analysis used a computerized format of CT that allowed a range of cognitive tasks to be repetitively practiced in a programmed format, which appeared to provide particular benefits to cognitive activities (e.g., planning and learning) that require cognitive effort to constantly or repeatedly process/hold/encode information. On the contrary, a decrease in performance on visual memory was observed following Game-based CT (McLaughlin et al., 2018). This finding can potentially be explained by the cognitive load interference theory, which suggests that an increase in verbal memory load may interfere with visual memory load (Cronin et al., 2020). Unlike verbal memory processes, which may benefit from the support of semantic and associative learning strategies, enhancing visual memory may necessitate more targeted and intensive training to evoke improvement in the relevant ability.

While meta-analyses did not reveal any significant benefits on attention/processing speed in healthy middle-aged adults following CT, qualitative synthesis found significant improvements in both healthy middle-aged adults (Anderson et al., 2013, 2014; Emch et al., 2019; Wolinsky et al., 2013, 2016) and menopausal population (Ballantyne et al., 2021; Pang & Kim, 2021). The meta-analysis results are also inconsistent with previous findings in healthy elderly (Ballesteros et al., 2014; Faust et al., 2020; H. K. Lee et al., 2020; Lee et al., 2013; Nouchi et al., 2012) and clinical populations (Åkerlund et al., 2013; Kim et al., 2022). A possible reason could be that middle-aged adults may not benefit as much as older adults, clinical populations, and menopausal women since some cognitive changes caused by older age, brain injury, or climacteric influences are more sensitive to CT in comparison to mild cognitive symptoms presented in healthy midlife. Moreover, processing speed may be particularly benefit from longer length of practice since some studies (Ackerman et al., 2010; McLaughlin et al., 2018) included in the meta-analyses had relatively shorter training lengths, which varied from 4 to 6 weeks, than those included in the qualitative synthesis (Anderson et al., 2013, 2014; Emch et al., 2019) that had an average of 8 weeks training length.

No significant benefit was found on language following 4–6 weeks’ CT from meta-analysis, while qualitative synthesis suggested significant benefits in menopausal population when following a 12-week CT (Pang & Kim, 2021). Decreased verbal fluency performance caused by the change of endogenous estrogen level during menopause transition may lead to the unique benefit of CT in menopausal women (Gurvich & Thomas, 2021; Laughlin et al., 2010). Moreover, the significant positive relationship of language performance with the total training length from meta-regression indicated that the benefits of CT for verbal fluency may emerge only after sufficient length of training.

### Effect of Training Types on Cognition

#### Training Content and Paradigm

General CT (Anderson et al., 2013, 2014; Corbett et al., 2015), Gamed-based CT, Working Memory Training (Emch et al., 2019), and Speed of Processing Training (Wolinsky et al., 2013, 2016) all utilized a computerized platform to train cognitive tasks but the training effects varied based on different content and paradigms. Working Memory Training (Emch et al., 2019; Felton et al., 2019; George et al., 2020) features a series of sequence recall or n-back tasks that usually adopts an adaptive paradigm by increasing or decreasing inter-stimulus interval based on participant’s performance in previous trials to adjust for training difficulties which thus optimize the training progress. Speed of Processing Training adopts a similar adaptive procedure by progressively decreasing the exposure time of stimulus (Wolinsky et al., 2013, 2016). Past research has indicated that adaptive procedures in interventions could confer greater benefits on sustained attention and information processing (Brehmer et al., 2012; Takeuchi & Kawashima, 2012), which may explain the significant benefits of both trainings on executive function and processing speed.

General CT shares a similar adaptive paradigm but consists of a more diverse training content and modes that target multiple cognitive domains, which may explain the significant improvements on various cognitive domains, including executive function, attention/processing speed, verbal memory, and working memory identified in qualitative synthesis (Anderson et al., 2013, 2014; Corbett et al., 2015; Roheger et al., 2020a, b). The auditory stimuli involved in one of the included General CT may also explain the substantial gains on multiple domains (Anderson et al., 2013). Auditory-based intervention was identified as a more efficacious format of CT to improve brain plasticity (e.g., increased regional grey matter and functional connectivity) and cognitive function (e.g., inhibitory control, information processing, and logical memory) in comparison to visual-based intervention in healthy older adults (Kawata et al., 2022) and clinical population (Scoriels et al., 2022). Therefore, General CT containing auditory stimuli may offer a greater benefit on multiple cognitive domains.

Although Gamed-based CT (Ackerman et al., 2010; McLaughlin et al., 2018) included a range of games targeting diverse cognitive abilities (except reasoning) and an adaptive procedure to adjust for game difficulty, it failed to yield significant improvements on other cognitive domains including working memory, attention/processing speed, and verbal/visual memory. One potential explanation could be that healthy adults improve their performance on the video games offered by the single platform (i.e., *The Big Brain Academy*), but this does not generalize to improve the cognitive abilities assessed as outcome measures. Previous studies in healthy younger and older adults suggested that cognitive abilities, including working memory, task switching, reasoning, visual memory, and visuospatial attention, were significantly improved particularly when the training included a greater perceptual effect (e.g., first-person shooter action game), real-time gaming (e.g., real-time interaction between two or more players), or strategy-based gaming (e.g., game requires decision-making and situational awareness; Basak et al., 2008; Green & Bavelier, 2006). The lack of improvement may also be attributed to the unstructured training paradigm, as participants were allowed to freely choose the games to play during the training time rather than practicing on prescribed ones defined by researchers (McLaughlin et al., 2018). Thus, future studies could explore a more structured Game-based CT that incorporates greater perceptual elements and use of strategies.

Both Strategy-based CT (Chapman et al., 2015; Pang & Kim, 2021; Unkenstein et al., 2017) and Cognitive Remediation (Ballantyne et al., 2021) utilized a non-computerized training format where participants were trained through a series of on-site in-group sessions. While self-reported (i.e., subjective) improvements were reported in verbal memory and attention/processing performances following the training, there was no improvement in objective, neuropsychological measures of working memory, verbal memory, attention/processing speed, and visual memory. It is possible that the group discussions, didactics, daily cognitive strategies, and lifestyle skills involved in Strategy-based CT and Cognitive Remediation may play unique roles in improving cognitive confidence during daily life. This may be particularly relevant for healthy middle-aged adults whose neuropsychological performance is likely to be in “normal” range.

Significant improvements on objective measures of executive function were observed following the Strategy-based CT and involved in-depth integrated reasoning and innovative thinking skills with respect to high-level cognitive control of complex information (Chapman et al., 2015). This study incorporated functional neuroimaging and found increased cerebral blood flow in the central executive network following Strategy-based CT, suggesting this type of training has the capacity to change brain function. Improvements on verbal fluency tasks were found following the Strategy-based CT (Pang & Kim, 2021) that consisted only of calendar training (i.e., daily practice of events tracking and diary writing). It is possible that calendar training promotes idea generation, verbal writing, and semantic practices that also underpin verbal fluency performance. Verbal fluency was significantly related to individual’s literacy and education levels at baseline, as well as the strategies one’s used during the assessment (da Silva et al., 2004; Mathuranath et al., 2003). Therefore, future interventions targeting verbal fluency may need to consider more semantic-focused training tasks as well as considering baseline literacy and education.

#### Training Parameters and Maintenance Effects

Training parameters (i.e., length, frequency, and booster sessions) are also likely to contribute to the efficacy of the CT. From the meta-analysis, for Game-based CT that adopted the same content and platform (i.e., *The Big Brain Academy*), a significant improvement was found on executive function and language performance following longer training length (i.e., 6 weeks; McLaughlin et al., 2018), but not shorter training length (i.e., 4 weeks; Ackerman et al., 2010). Strategy-based CT, involving calendar training or reasoning training, showed significant benefits on executive function, language, and verbal memory following a minimum training period of 12 weeks (Chapman et al., 2015; Pang & Kim, 2021). Moreover, Speed of Processing Training with single booster sessions conferred greater benefits on cognitive tasks than training without booster sessions (Wolinsky et al., 2013, 2016). In general, the minimum requirement of training length associated with a cognitive benefit was five training sessions across 4 weeks (Ackerman et al., 2010); however, there were significant variations in training duration and frequency across different types of training. Further research is required to explore the minimum dosage of training that can deliver significant benefits on different cognitive domains.

Due to the unavailability of data—specifically, the lack of immediate post-data for comparison with follow-up data, or the limited number of studies using randomized controlled trials in a specific cognitive domain—five primary studies involving follow-up assessments were not included in the meta-analysis. These studies comprised three randomized controlled trials (Anderson et al., 2013; George et al., 2020; Wolinsky et al., 2013) and two single-case studies (Ballantyne et al., 2021; Unkenstein et al., 2017). From the qualitative analysis, a maintenance effect (which was measured at various times from 1-month to 1-year post-CT) was observed for verbal memory, working memory, and attention/processing speed tasks following Working Memory Training (George et al., 2020), Speed of Processing Training (Wolinsky et al., 2013, 2016), and General CT (Anderson et al., 2013, 2014), as well as on self-reported cognitive confidence following Strategy-based CT (Unkenstein et al., 2017) and Cognitive Remediation (Ballantyne et al., 2021). This demonstrates the potential longer-term benefits that CT might offer particularly when CT involves adaptive paradigms, strategies education, longer training length (up to 12 weeks), and booster sessions. Therefore, future research should investigate the most efficacious parameters for different types of CT as well as include follow-up assessment to evaluate the maintenance effects.

### Generalizability of CT and Population Characteristics

Mood was reported as a secondary outcome in four studies (Ballantyne et al., 2021; McDougall & House, 2012; Pang & Kim, 2021; Unkenstein et al., 2017). Only one of these studies identified an improvement in mood following CT. A reduction in anxiety was reported following 18 sessions of Game-based CT, which was found to be associated with improved performance on executive function (McLaughlin et al., 2018). This is somewhat consistent with one study reporting a significant association between improved perceived quality of life and perceived cognitive functioning following Game-based CT in older adults (McDougall & House, 2012), suggesting that Game-based CT may improve perceptions of quality of life and reduce mood disturbances by enhancing cognitive self-efficacy. In contrast to these findings, there were no significant improvements observed in mood, self-esteem, or menopausal symptoms following Strategy-based cognitive training (Pang & Kim, 2021; Unkenstein et al., 2017) or Cognitive Rehabilitation (Ballantyne et al., 2021). It is possible that the generalizability of CT may be limited by certain types of training, which require further research. To be noted, these studies did not report any improvements in objective cognition, and this may be a key factor in failing to detect improvements in mood. While this review did not specifically focus on mood and self-esteem as an outcome, they are often closely related to cognition (Curvis et al., 2018; Gotlib & Joormann, 2010) and future research in CT may benefit from including mood, quality of life, self-efficacy, and specifically looking at the associations between these measures and cognition before and after training.

The population health-related characteristics were mostly specified within the included studies, with participants screened for any psychiatric disorders or health conditions that could affect cognition. However, other health-related conditions, such as cardiovascular diseases, sleep disturbances, and hypertension, were not examined. These conditions may influence the generalizability of CT, particularly if self-reported measures on quality of life or self-esteem were considered (Djärv et al., 2012; Hedayati, 2015). While one of the original aims of this review was to determine the effects of CT for menopause-related cognitive symptoms, only three studies specifically focused on middle-aged women during the menopausal transition. The findings of these studies revealed improvements in verbal fluency tasks and self-reported verbal learning performance following Strategy-based CT (Pang & Kim, 2021; Unkenstein et al., 2017), as well as increased confidence in verbal learning, executive function, and attention following Cognitive Remediation (Ballantyne et al., 2021). However, given the common experience of brain fog during menopause, there could be significant potential for CT specifically for this population.

## Strengths and Limitations

This systematic review and meta-analysis adopted a comprehensive definition of cognitive training (CT), and an approach combining qualitative synthesis with meta-analysis, which allowed diverse types of CT to be both quantitively and narratively discussed with respect to a range of cognitive domains presenting by either near- or far-transfer outcomes in midlife. This is the first review to synthesize the evidence exploring cognitive training in a menopause population.

Due to data unavailability and limited study numbers, studies looking at Speed of Processing Training (Wolinsky et al., 2013, 2016) and Cognitive Remediation (Ballantyne et al., 2021) were not included in the meta-analysis. Subgroup analysis based on population (e.g., healthy middle-aged adults and healthy menopausal women), training types (e.g., General CT and Game-based CT), and maintenance effect was also not possible due to limited studies. In addition, since the GRADE tool suggested low to moderate certainty of evidence for most domains, which could be attributed to the observed limited number of comparisons and high heterogeneities of training parameters, the meta-analysis results might be statistically underpowered.

Another limitation of the current review is the high level of heterogeneity within certain cognitive domains. This variability may result from the use of different types of CT and different neuropsychological measures. Therefore, future research should consider conducting sensitivity analyses when the number of included studies allows or examine the construct validity of each test and explore the correlations between these measures.

## Conclusions

Transfer improvement of CT was observed on multiple cognitive domains in healthy midlife adults. Specifically, CT (e.g., Working Memory Training, Speed of Processing Training, General CT, and Game-based CT) adopting adaptive paradigms, utilizing computerized/auditory-based platforms, and having longer training lengths and booster sessions demonstrates benefits on executive function, processing speed, working memory, and learning tasks. Strategy-based CT involving in-depth cognitive strategies and reasoning skills shows particular benefits on executive function. Verbal fluency is specifically sensitive to Strategy-based CT that encompasses written and semantic practice. Subjective cognition received specific gains from Strategy-based CT and CR in menopausal women, suggesting the possibility of using CT as a compensatory approach to ameliorate menopausal-related cognitive change. Overall, CT may offer promise to promote cognitive health and brain health during the midlife period.

## Supplementary Information

Below is the link to the electronic supplementary material.

Supplementary file1 (DOCX 1891 KB)

## Data Availability

The data that support the findings of this study are available from the corresponding author, C.G., upon reasonable request.
