# Supplementary material for: Cognitive Training During Midlife: A Systematic Review and Meta-Analysis
Source: Neuropsychol Rev. 2024 Sep 5;35(3):427–48. doi: 10.1007/s11065-024-09649-z (PMC12602558; doi:10.1007/s11065-024-09649-z)

| **eTable 1.** Inclusion and Exclusion Criteria of Studies for Narrative Synthesis and Meta-analysis. | | |  |
| --- | --- | --- | --- |
| **Analysis Type** | **Criteria** | **Inclusion** | **Exclusion** |
| Narrative Synthesis | Population | a) healthy middle-aged adults’ using that broad definition as well as any additional inclusion and exclusion criteria outlined in each included original study (e.g. if the original study also excluded psychiatric symptoms or disorders)  b) age range OR mean age -1 standard deviation > 40 AND mean age +1 standard deviation < 66 at least in one intervention group | a) animals  b) diagnosed with clinical disease, health condition or mental/psychotic disorder that may impact cognitive functioning (such as head injury, HIV, diagnosed neurological disorders, etc.)  c) survivors of diseases/medical conditions |
|  | Design | a) observational/retrospective studies looking at the relationship between intervention and intervention outcomes  b) RCTs/cross-over/repeated-measure/longitudinal involving interventions  c) include two or more assessment time points (e.g., pre, post, follow-ups) | a) review, reports, protocols, news, etc. |
|  | Outcome | a) include objective measure of at least one cognitive domain or a global cognitive score at behavioral level  b) include subjective measure of at least one cognitive domain or a global cognitive score | a) Studies assessed cognition using neurological instrument only (such as fMRI, EEG etc.)  b) Studies assessed psychological outcome (such as depression, anxiety, etc.) without any cognitive outcome |
|  | Intervention | a) at least one intervention group involves pure cognitive training | a) intervention involves other non-pharmacological component that different with cognitive training (e.g., CBT, yoga, physical exercise or mindfulness-based training) |
|  | Language | English | a) Non-English |
| Meta-analyses | Design | RCTs | a) Single-case  b) Observational/retrospective |
|  | Data Availability | a) mean and standard deviation at baseline, AND  b) mean and standard deviation at immediate post, AND  c) sample size at immediate post, AND  d) at lease two studies were included in the meta-analysis of specific outcome | a) only one type of statistic is available  b) only one study was included in the meta-analysis of specific outcome  c) only one time-point statistic is available  d) identified as outliers |

RCTs, Randomized control trials; fMRI, Functional magnetic resonance imaging; EEG, Electroencephalography; CBT, Cognitive behavioral therapy

| **eTable 2.** Risk of Bias Assessment for Included Studies in Qualitative Synthesis. | | | | | | | | | | |
| --- | --- | --- | --- | --- | --- | --- | --- | --- | --- | --- |
| **Study** | **Cross-over/RCTs (Retrospective/Single-case Design/Quasi-experimental)** | | | | | |  | | | **Overall** |
|  | **Randomization Process (Measurement of Exposure)** | **Period & Carryover Effects (Selection of Participants)** | **Deviations from Intervention** | **Missing Outcome** | **Outcome Measurement** | **Selection of Reported Results** | | **(Confounding)** | |  |
| Ackerman et al., 2010 | Low | Low | Low | Low | Low | Low | | | NA | Low Risk |
| McLaughlin et al., 2018 | Some Concerns | Some Concerns | Low | Low | Low | Low | | | NA | Some Concerns |
| Corbett et al., 2015 | Low | NA | Low | Low | Low | Low | | | NA | Low Risk |
| Roheger et al., 2020_1 | Low | NA | Low | Low | Low | Low | | | NA | Low Risk |
| Roheger et al., 2020_2 | Low | NA | Low | Low | Low | Low | | | NA | Low Risk |
| Felton et al., 2019 | Low | NA | Low | Low | Low | Low | | | NA | Low Risk |
| Namratha, Bajaj & Bhat, 2017 | Low | NA | Some Concerns | Low | Low | Low | | | NA | Some Concerns |
| Mridula, et al., 2017 | Low | NA | Some Concerns | Low | Low | Low | | | NA | Some Concerns |
| George, Bajaj & Bhat, 2020 | Low | NA | Some Concerns | Low | Low | Low | | | NA | Some Concerns |
| Emch, et al., 2019 | Low | NA | Low | Low | Low | Low | | | NA | Low Risk |
| Wolinsky et al., 2013 | Some Concerns | NA | Some Concerns | Low | Low | Low | | | NA | Some Concerns |
| Wolinsky et al., 2016 | Some Concerns | NA | Some Concerns | Low | Low | Low | | | NA | Some Concerns |
| Anderson et al., 2013 | Low | NA | Low | Low | Low | Low | | | NA | Low Risk |
| Anderson et al., 2014 | Low | NA | Low | Low | Low | Low | | | NA | Low Risk |
| Chapman et al., 2015 | Low | NA | Low | Low | Low | Low | | | NA | Low Risk |
| Bonnechère et al., 2021 | Some Concerns | Some Concerns | Low | Low | Low | Low | High | | | High Risk |
| Unkenstein et al., 2017 | Low | Low | Low | Low | Low | Low | Low | | | Low Risk |
| Pang & Kim, 2021 | Low | Low | Low | Low | Low | Low | Low | | | Low Risk |
| Ballantyne et al., 2021 | Low | Low | Low | Some Concerns | Low | Low | Some Concerns | | | Some Concerns |
| Revised Cochrane Risk-of-Bias (RoB2) tool for randomized control trials, RoB2 Additional Considerations for crossover trials, Risk of Bias in Non-randomized Studies of Exposure (ROBINS-E) for retrospective study, and Risk of Bias in Non-randomized Studies of Interventions (ROBINS-I) for single-case and quasi-experimental study.  NA, Not Applicable. | | | | | | | | | | |

| **eTable 3.** Certainty of evidence by Grading of Recommendations Assessment, Development, and Evaluation (GRADE) approach. | | | | | | | | | | | | |
| --- | --- | --- | --- | --- | --- | --- | --- | --- | --- | --- | --- | --- |
| **Cognitive domain** | **Certainty assessment** | | | | | | | **No. of patients** | | **Effect** | **Certainty** | **Importance** |
|  | **No. of cases** | **Study design** | **Risk of bias** | **Inconsistency** | **Indirectness** | **Imprecision** | **Other considerations** | **Cognitive training** | **Active or**  **passive control** | **Absolute (95% CI)** |  |  |
| Executive function | 10 | RCT | not serious | very serious^a^ | not serious | not serious | none | 1237 | 546 | SMD 0.48 SD higher (0.08 higher to 0.87 higher) | ⨁⨁◯◯ Low | CRITICAL |
| Processing speed | 9 | RCT | not serious | serious^a^ | not serious | not serious | none | 1205 | 534 | SMD 0.01 SD higher (0.10 lower to 0.11 higher) | ⨁⨁⨁◯ Moderate | CRITICAL |
| Language | 6 | RCT | not serious | very serious^a^ | not serious | not serious | none | 143 | 143 | SMD 0.16 SD higher (0.4 lower to 0.72 higher) | ⨁⨁◯◯ Low | CRITICAL |
| Verbal memory | 8 | RCT | not serious | not serious | not serious | not serious | none | 1147 | 454 | SMD 0.22 SD higher (0.11 higher to 0.33 higher) | ⨁⨁⨁⨁ High | CRITICAL |
| Visual memory | 2 | RCT | serious^b^ | not serious | not serious | not serious | none | 14 | 14 | SMD 0.97 SD lower (1.77 lower to 0.17 lower) | ⨁⨁⨁◯ Moderate | CRITICAL |
| Working memory | 7 | RCT | not serious | serious^a^ | not serious | not serious | none | 1164 | 469 | SMD 0.16 SD higher (0.05 higher to 0.26 higher) | ⨁⨁⨁◯ Moderate | CRITICAL |
| Grade Definition (Schünemann et al., 2013)  High certainty: Further research is very unlikely to change our confidence in the estimate of effect.  Moderate certainty: Further research is likely to have an important impact on our confidence in the estimate of effect and may change the estimate.  Low certainty: Further research is very likely to have an important impact on our confidence in the estimate of effect and is likely to change the estimate.  Very low certainty: Any estimate of effect is very uncertain.  RCT, Randomized Control Trial; CI, Confidence Interval; SMD, Standardized Mean Difference; SD, Standard Deviation; No., Number.  ^a.^ Significant heterogeneity  ^b.^ Cases from single primary study | | | | | | | | | | | | |

**eFig. 1** Funnel plots for attention/processing speed with significance level at *P* < .05. Dotted vertical line indicates the line of no effect.

**
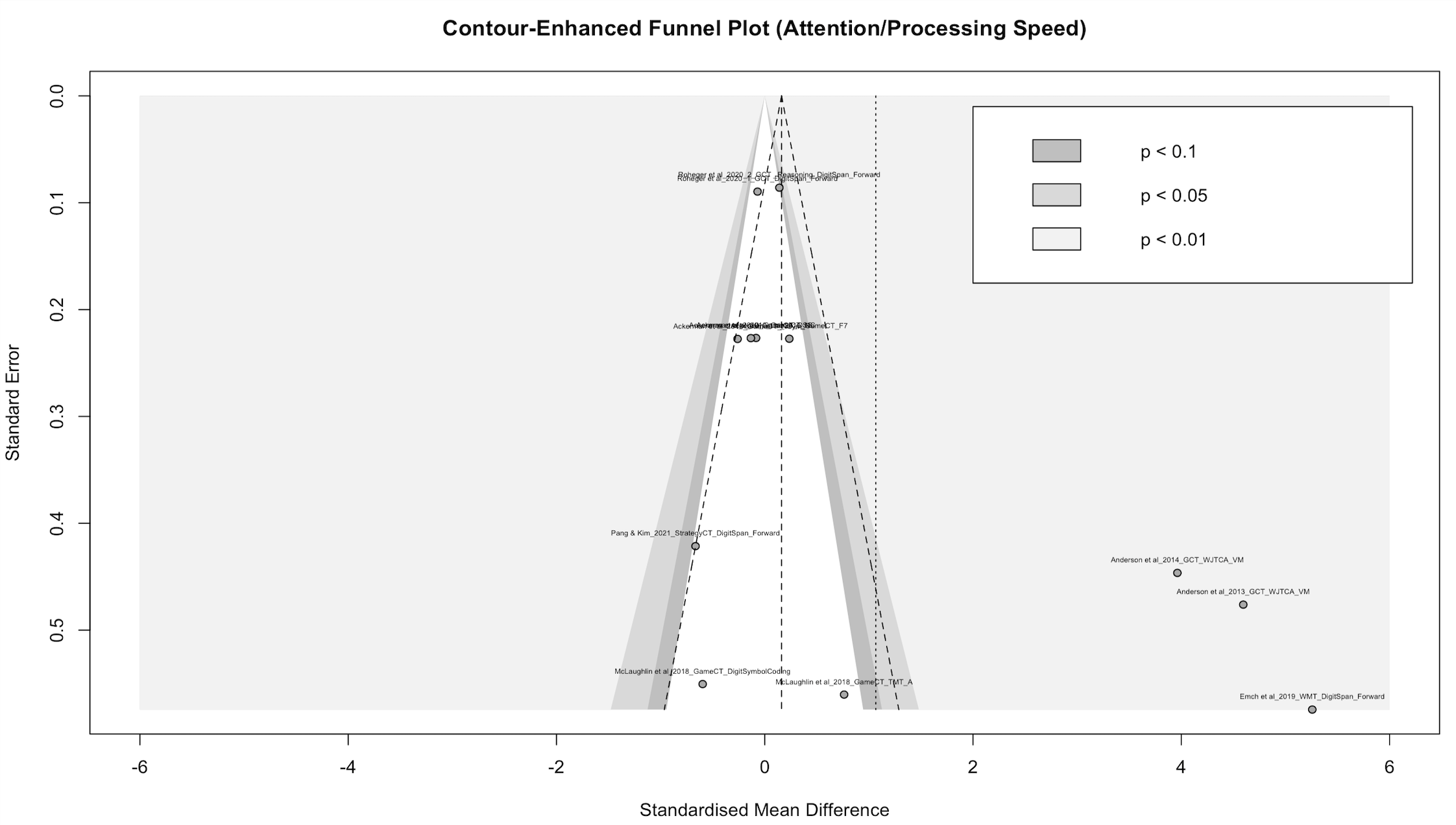
**

**eFig.2** Funnel plots for language with significance level at *P* < .05. Dotted vertical line indicates the line of no effect.

**
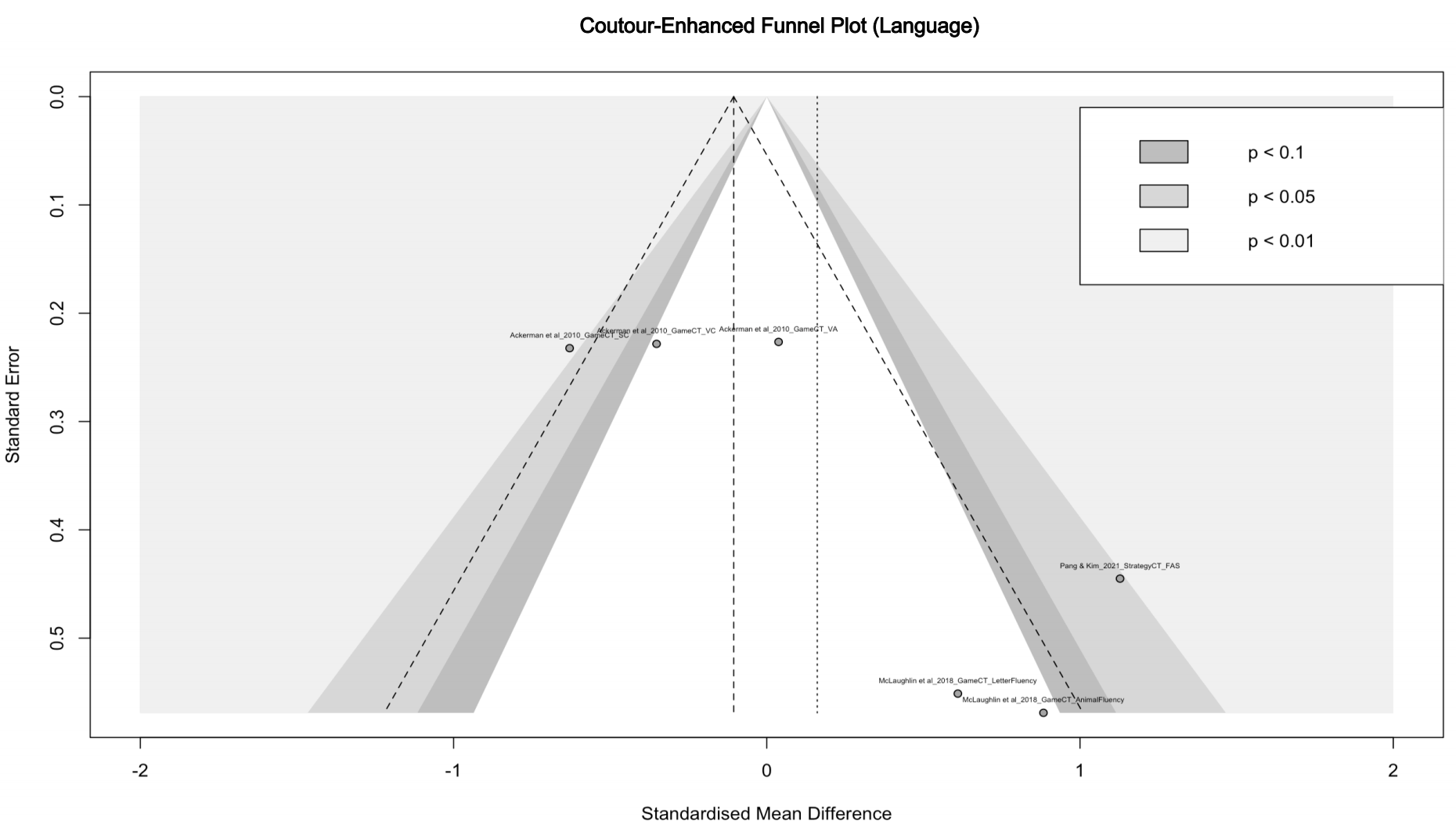
**

**eFig.3** Funnel plots for verbal memory with significance level at *P* < .05. Dotted vertical line indicates the line of no effect.

**
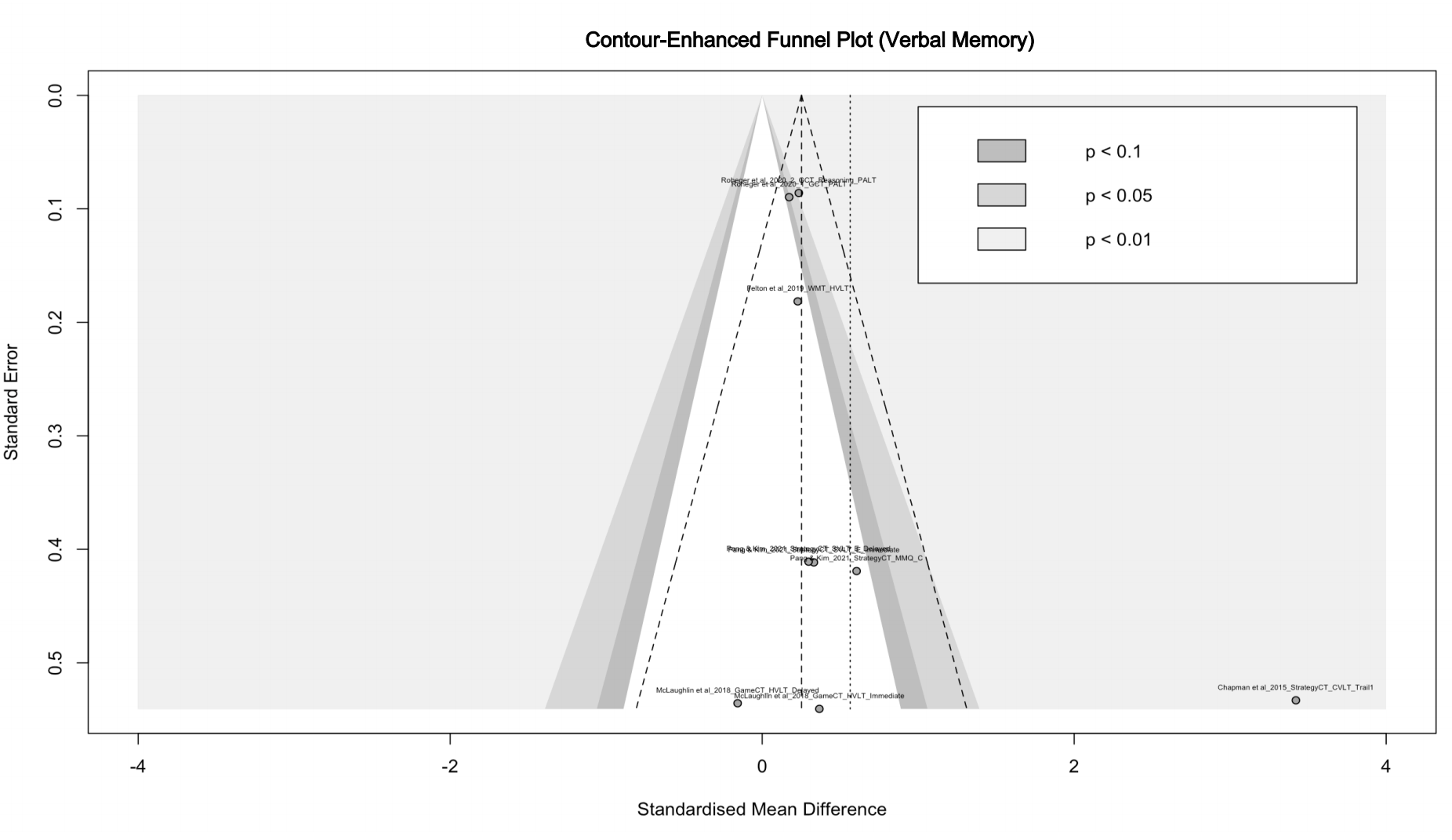
**

**eFig.4** Funnel plots for visual memory with significance level at *P* < .05. Dotted vertical line indicates the line of no effect.

**
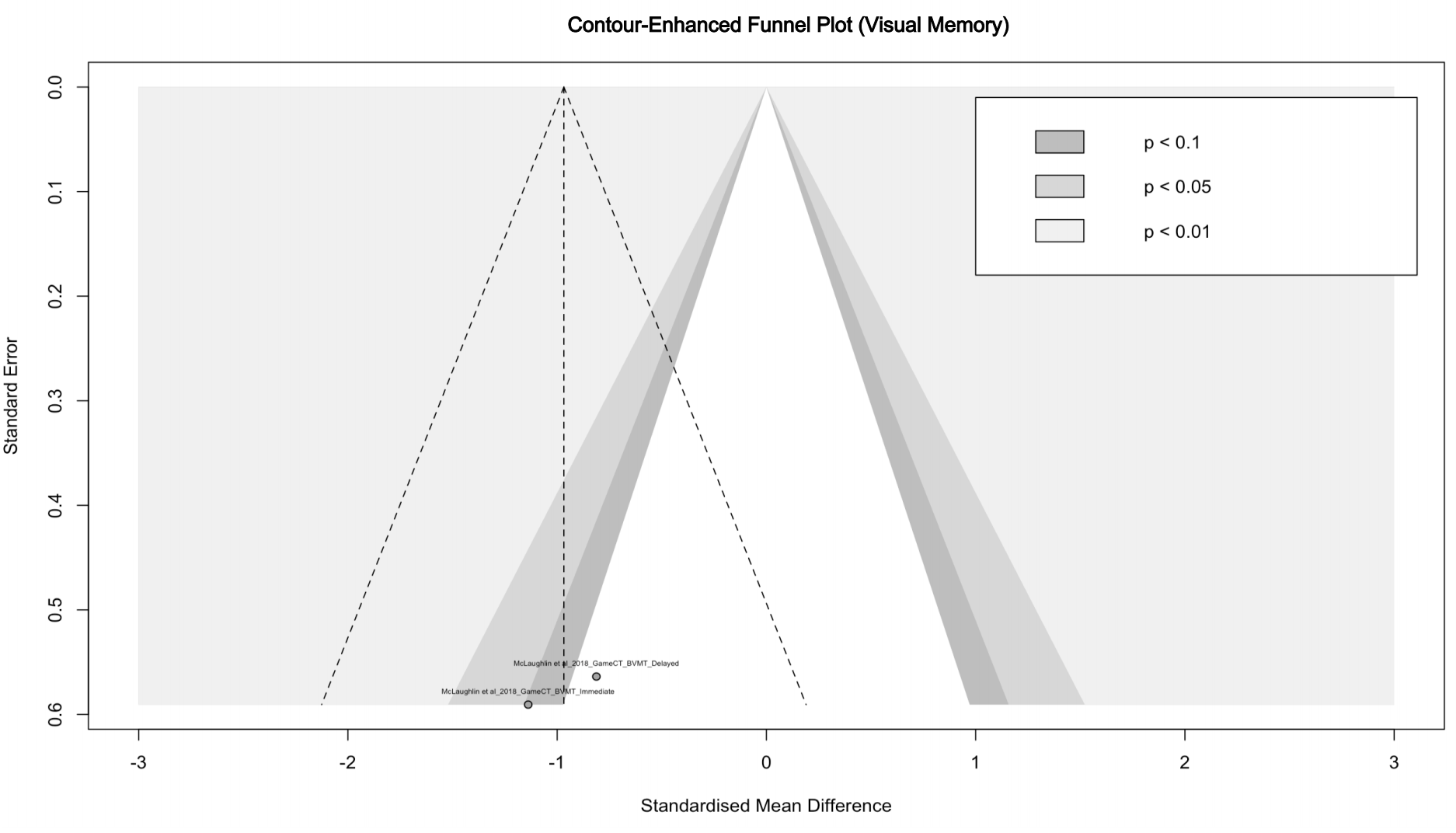
**

**eFig.5** Funnel plots for working memory with significance level at *P* < .05. Dotted vertical line indicates the line of no effect.


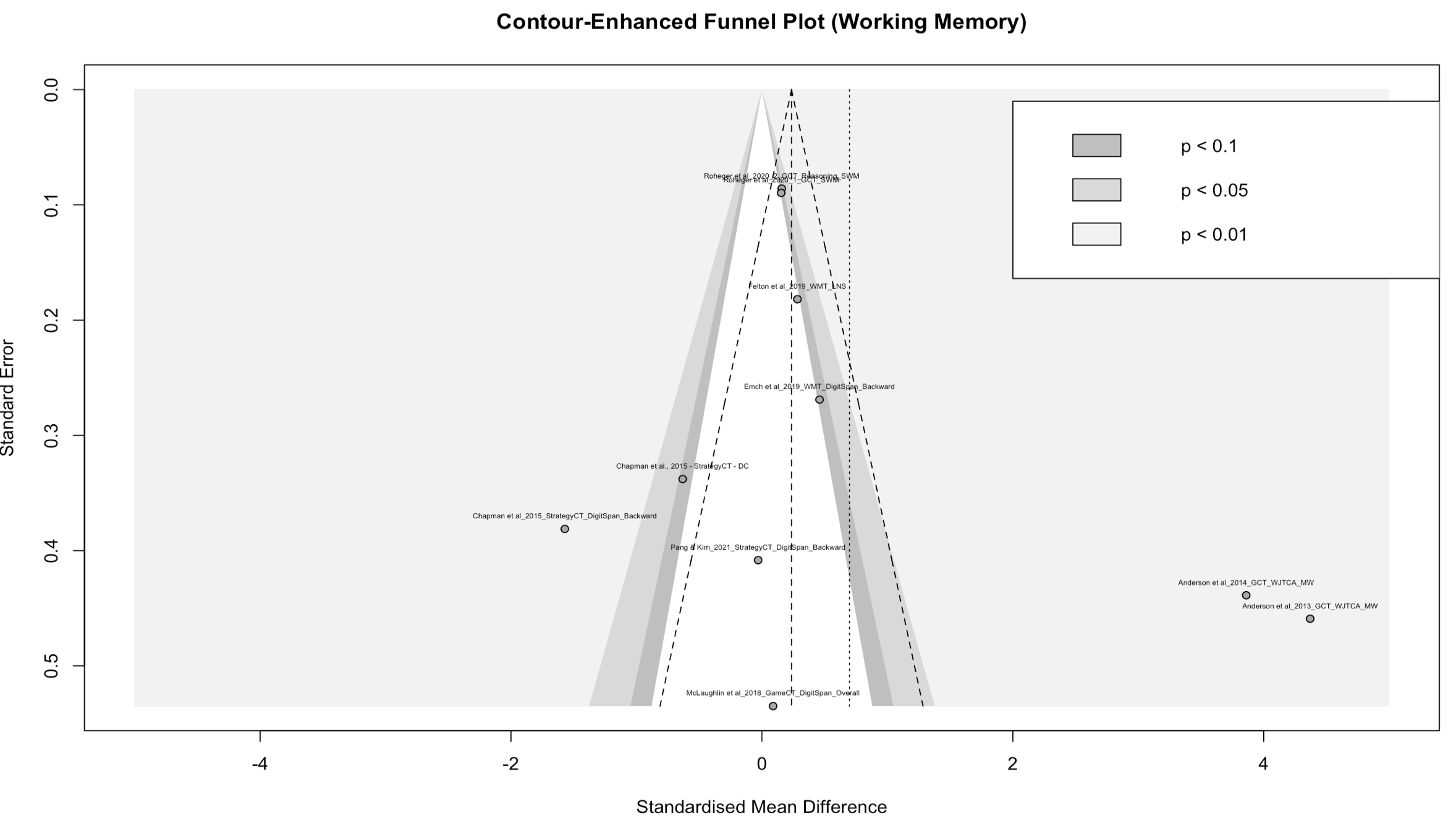


**eFig.6** Funnel plots for executive function with significance level at *P* < .05. Dotted vertical line indicates the line of no effect.


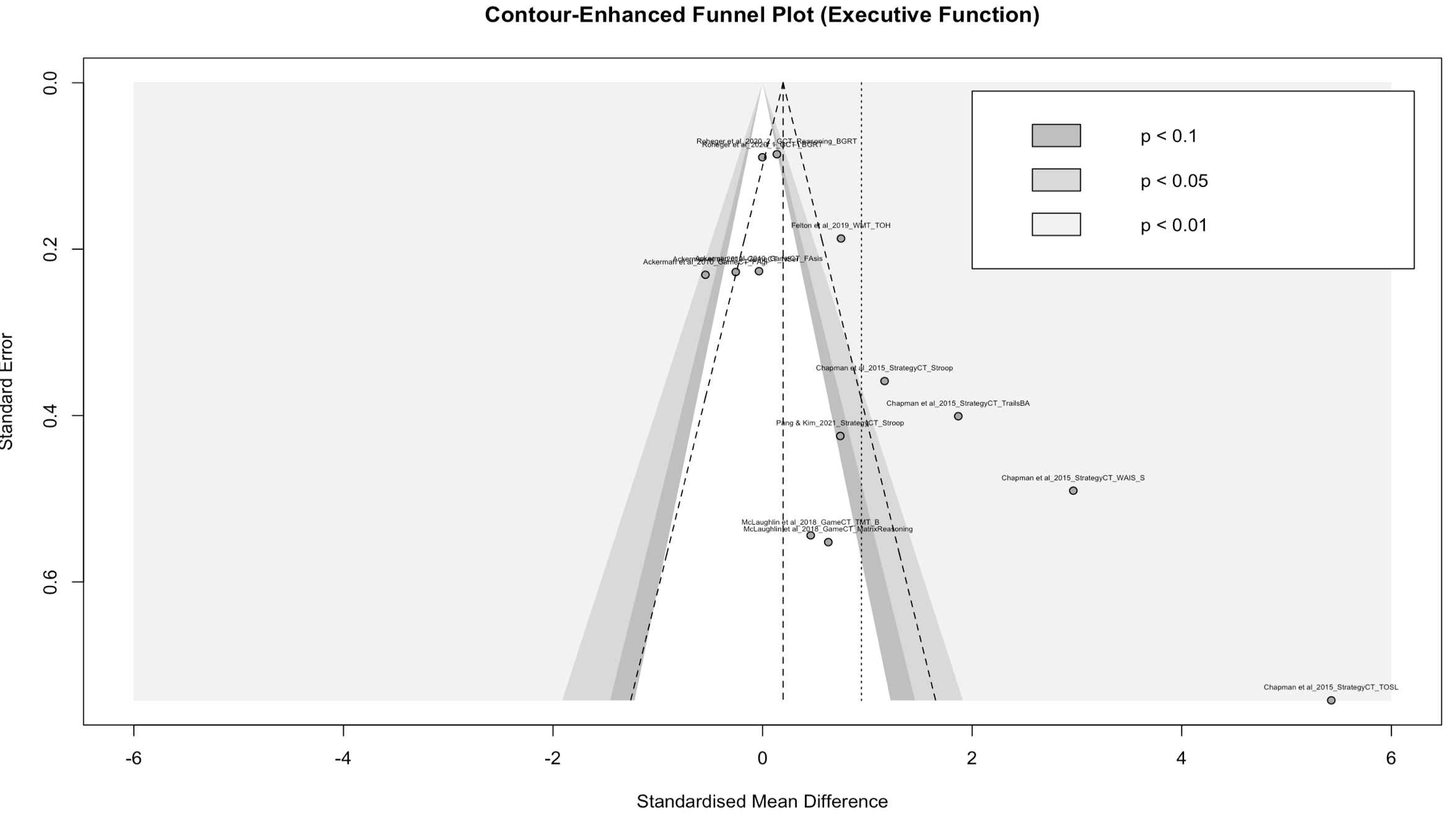

Supplement: Supplementary file 1 — Supplementary file1 (DOCX 1891 KB) [file 11065_2024_9649_MOESM1_ESM.docx]
